# Changes in Renal Function in Patients with Recurrence of Atrial Arrhythmia after an Initial Catheter Ablation

**DOI:** 10.1155/2022/6923377

**Published:** 2022-01-31

**Authors:** Youmei Shen, Hongwu Chen, Gang Yang, Weizhu Ju, Fengxiang Zhang, Kai Gu, Chang Cui, Mingfang Li, Minglong Chen

**Affiliations:** Division of Cardiology, The First Affiliated Hospital of Nanjing Medical University, Nanjing 210029, China

## Abstract

**Background:**

Impaired renal function and atrial fibrillation (AF) can form a vicious cycle. Although there have been reports on improved renal function in patients who undergo successful AF ablation, renal function in patients with recurrence of AF has not been studied separately. We explored the changes in renal function in recurrent AF patients after catheter ablation with mild renal dysfunction and the influencing factors.

**Methods:**

We retrospectively recruited nonvalvular AF (NVAF) patients with mildly impaired renal function admitted for catheter ablation and readmitted due to recurrence of AF. The estimated glomerular filtration rate (eGFR) was calculated before the index procedure and during readmission. *△*eGFR was defined as the difference between eGFR _readmission_ and eGFR _baseline_. The same calculation applied for *△*CHA_2_DS_2_-VASc score. The primary endpoint was improved renal function (△eGFR >0) after AF catheter ablation in patients with atrial arrhythmia recurrence.

**Results:**

A total of 132 NVAF patients were included in this study. The mean eGFR at readmission was significantly increased compared with the eGFR at baseline before the index ablation procedure (81.5 ± 1.1 vs. 78.0 ± 0.7 ml/min/1.73 m^2^, *P* < 0.001). The multivariable Cox regression analysis showed that a lower *△*CHA_2_DS_2_-VASc score (HR: 0.42, *P*=0.003) and paroxysmal recurrent atrial arrhythmia (HR: 2.97, *P*=0.001) were associated with better renal function.

**Conclusion:**

In NVAF patients with mildly impaired renal function, even those with recurrence after the initial catheter ablation, we observed improvements in renal function, which was associated with a lower *△*CHA_2_DS_2_-VASc score and paroxysmal recurrent arrhythmia.

## 1. Introduction

Atrial fibrillation (AF) is one of the most common arrhythmias in adults [1]. As a degenerative disease, AF is expected to be a growing health burden with the changing demographics and increased life expectancy across the globe [2–4]. Due to the irregular ventricular rate and cardiac thrombus, in addition to increasing risk of stroke, AF has a profound and comprehensive influence on multiple organs, such as leading to decrements in cardiac function, cognitive function, and renal function [5–8]. Moreover, symptomatic AF reduces patient quality of life.

Due to the limited effectiveness of antiarrhythmic drugs, catheter ablation (CA) has been considered to be a promising solution to restore sinus rhythm, reduce the AF burden, and even extend survival [9–17]. Successful sinus rhythm restoration through CA has been known to be associated with reduced heart failure or death and improved cognitive function, quality of life, and renal function [12, 18–20]. Takahashi et al. reported improvements in renal function with successful CA after a follow-up of more than 1 year [19]. Wang et al. reported improvements in renal function after repeated CA in patients with long-standing AF [21]. Park et al. reported better renal function in patients who maintained sinus rhythm after more than 5 years of follow-up [22]. A number of predictors for worsening renal function have been identified, including the CHA_2_DS_2_-VASc score, preexisting diabetes mellitus, and AF recurrence. However, few studies have focused on changes in renal function in AF patients with arrhythmia recurrence.

The objective of this study was to investigate the changes in the estimated glomerular filtration (eGFR) in nonvalvular atrial fibrillation (NVAF) patients with recurrence after radiofrequency catheter ablation (RFCA).

## 2. Methods

### 2.1. Study Design

This was a retrospective observational study of hospitalized patients using their administrative data. This study was conducted according to the Declaration of Helsinki and institutional guidelines. The requirement for patient consent was waived because of the retrospective nature of this study. Routinely collected health data were analyzed in this study.

### 2.2. Study Population

We reviewed the electronic health records to identify AF patients with at least two admissions to the First Affiliated Hospital of Nanjing Medical University between January 2012 and June 2019. NVAF was confirmed according to the International Classification of Disease- (ICD-) 10 diagnostic code I48.0–48.4, I48.9. Additional selection criteria were AF catheter ablation performed during the 1^st^ admission and rehospitalization due to AF recurrence and mildly impaired renal function at the 1^st^ admission. The exclusion criteria were as follows: less than 3 months between the two admissions, and diagnosis of chronic kidney disease before the index AF catheter ablation.

All clinical characteristics before the index procedure and during readmission were collected. The Chronic Kidney Disease Epidemiology Collaboration (CKD-EPI) equation was used to calculate the eGFR to assess renal function [23].

### 2.3. Catheter Ablation Procedure

Patients with paroxysmal AF routinely underwent circumferential pulmonary vein isolation (CPVI). In nonparoxysmal AF patients, the STABLE-SR strategy was used to target the fibrotic areas; this strategy has been well described and was previously published [24]. In brief, all nonparoxysmal AF patients underwent CPVI and cavotricuspid isthmus (CTI) ablation. Then, we performed electrical cardioversion to restore sinus rhythm. An A-Focus catheter (St. Jude Medical, St Paul, MN) was used to generate a high-density bipolar map of the left atrium. In the low-voltage zone, all tissues were homogenized. In the transitional zones, complex electrograms were eliminated. Dechanneling line was performed if necessary. If AF continued after cardioversion, we performed linear ablation, including the left atrium roof line and the mitral isthmus line. Ablation of complex fractional electrograms was performed to terminate AF if necessary.

### 2.4. Primary Endpoint

Improved renal function after AF catheter ablation with atrial arrhythmia recurrence was defined as *eGFR*_readmission_ − *eGFR*_baseline_ >0.

### 2.5. Definitions

According to the National Kidney Foundation Kidney Disease Outcomes Quality Initiative (NKF KDOQI), mild renal dysfunction was defined as an eGFR of 60 to 90 ml/min/1.73 m^2^ [25].

The changes in CHA_2_DS_2_-VASc score and renal function are presented by the absolute change in the following two variates:

- △CHA_2_DS_2_-VASc score = CHA_2_DS_2_-VASc score _readmission_ − CHA_2_DS_2_-VASc score _baseline_
- △eGFR = eGFR _readmission_ − eGFR _baseline_

The blank period was defined as the first 3 months after the RFCA procedure. The recurrence of paroxysmal atrial arrhythmia after RFCA was defined as atrial arrhythmia, including atrial fibrillation, atrial flutter, and atrial tachycardia, lasting longer than 30 s but less than 1 week. The recurrence of persistent atrial arrhythmia after RFCA was defined as any atrial arrhythmia lasting longer than 1 week.

We classified the pattern of arrhythmia change before and after the index ablation into 4 groups: paroxysmal AF to paroxysmal arrhythmia recurrence (Pa to Pa), paroxysmal AF to persistent arrhythmia recurrence (Pa to Pe), persistent AF to paroxysmal arrhythmia recurrence (Pe to Pa), and persistent AF to persistent arrhythmia recurrence (Pe to Pe).

### 2.6. Statistical Analysis

Continuous variables are expressed as the mean ± SD or median and interquartile range (IQR) if nonnormally distributed. Categorial variables are presented as numbers (percentages). eGFR at baseline and after readmission were compared using paired *t*-tests. Comparisons of the changes in eGFR between recurrent arrhythmia groups were tested by one-way ANOVA. A Cox regression was used to identify possible factors predicting an improvement in eGFR after AF catheter ablation in patients with atrial arrhythmia recurrence. The duration between the two admissions was used as the time scale in the Cox model. Variables with a *P* value <0.05 in the univariate analysis and age were included in the multivariable model. All data were analyzed by SPSS Statistics version 25.0 (IBM Corp. in Armonk, NY).

## 3. Results

**Figure 1:**
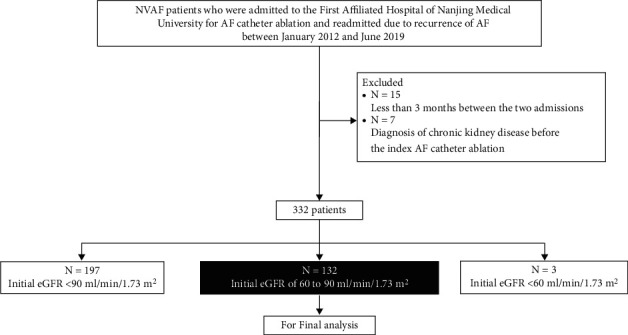
Flowchart depicting patient selection processes.

Between January 2012 and June 2019, a total of 332 patients were admitted to The First Affiliated Hospital of Nanjing Medical University for AF catheter ablation and readmitted due to recurrence of AF. Among them, 197 had an initial eGFR >90 ml/min/1.73 m^2^, 132 with eGFR of 60–90 ml/min/1.73 m^2^, and 3 with eGFR <60 ml/min/1.73 m^2^. In the present study, all 132 AF patients with an eGFR of 60–90 ml/min/1.73 m^2^ were finally analyzed.  Figure 1 depicts the study flowchart.

Table 1 summarizes the clinical characteristics at baseline during the initial admission. Among all the patients, 66.7% were men (*n* = 88) and 54.5% (*n* = 72) were diagnosed with paroxysmal AF. The mean age was 62.8 ± 0.7 years. The baseline CHA_2_DS_2_-VASc score was 1.7 ± 0.1.

**Figure 2:**
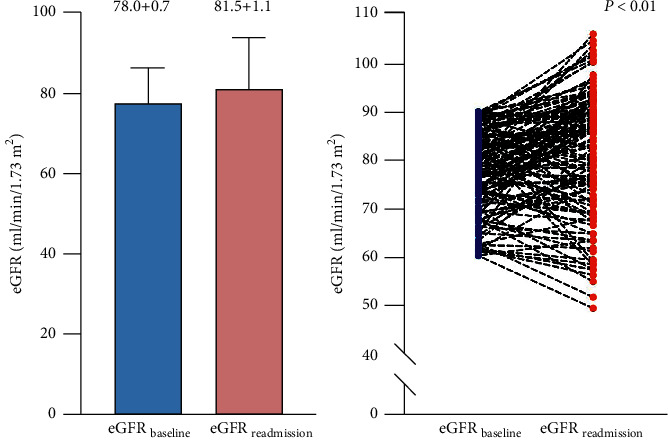
The mean change in and scatter plot of the estimated glomerular filtration rate (eGFR) values at baseline and during readmission.

The median duration between the two admissions was 11 months (IQR: 6–22 months). The *△*CHA_2_DS_2_-VASc score was 0.2 ± 0.0. The mean eGFR at readmission was significantly increased compared with the eGFR at baseline before the index RFCA procedure (81.5 ± 1.1 vs. 78.0 ± 0.7 ml/min/1.73 m^2^, *P* < 0.001), as shown in Figure 2. After all patients underwent successful CPVI, 94 (71.2%) patients showed paroxysmal atrial arrhythmia and the remaining 38 (28.8%) showed persistent atrial arrhythmia when readmitted due to recurrence.

**Figure 3:**
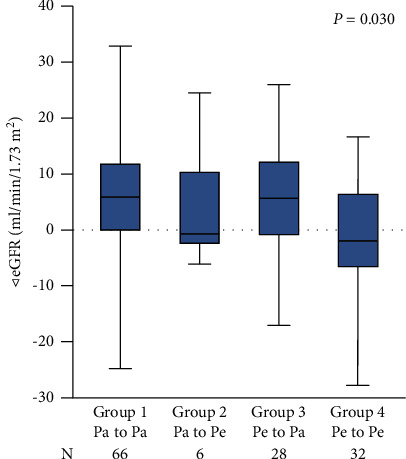
Median eGFR changes grouped by arrhythmia pattern changes.

**Figure 4:**
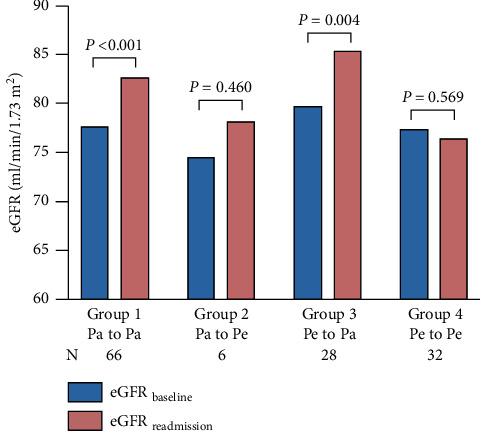
Different eGFR changes in each arrhythmia pattern change group.

Among the 4 groups classified based on patterns of arrhythmia change, the difference in *△*eGFR was statistically significant (*P*=0.030), as shown in Figure 3.  Figure 4 shows that patients with recurrence of paroxysmal atrial arrhythmia had better renal function outcomes regardless of whether they initially had paroxysmal or persistent AF (*P* < 0.001 and *P*=0.004, respectively).

In the univariate Cox regression, the *△*CHA_2_DS_2_-VASc score, left atrial diameter (LAD), and recurrent atrial arrhythmia type were significantly associated with changes in eGFR. Although not a significant factor in the univariate Cox regression, baseline age was still included in the multivariable analysis because age has always been a strong risk factor for renal dysfunction. As shown in Table 2, after adjusting for baseline age and the *△*CHA_2_DS_2_-VASc score, LAD, and recurrent atrial arrhythmia type, the multivariable Cox regression showed that a lower *△*CHA_2_DS_2_-VASc score (hazard ratio (HR): 0.42, 95% confidence interval (CI): 0.235–0.735, *P*=0.003) and paroxysmal recurrent atrial arrhythmia (HR: 2.97, 95% CI: 1.604–5.483, *P*=0.001) were associated with better renal function.

## 4. Discussion

By comparing the eGFR before RFCA to the eGFR during readmission for recurrence, we found that renal function could still be improved after RFCA in NVAF patients with a mildly decreased eGFR, even if they had recurrent atrial arrhythmia.

The main reasons why we focused on a selective group of AF patients who underwent ablation were as follows. First, AF patients with eGFR <60 mL/min/1.73 m^2^ had greater risk for periprocedural complications and AF recurrence, when compared with those with better renal function. Therefore, the reluctance to choose catheter ablation as a therapeutic strategy for AF patients with eGFR <60 mL/min/1.73 m^2^ is common in daily practice. As a matter of fact, totally, there were only 3 patients with eGFR <60 mL/min/1.73 m^2^ who underwent the initial catheter ablation and were readmitted due to AF recurrence in our center during the study period. This sample size was too small for statistical reporting. Second, the increase of eGFR level in patients with normal renal function may not be of great importance. Thus, we selected AF patients with mildly impaired renal function to study.

Among general populations, the annual decline in eGFR is between 0 and 1 ml/min/1.73 m^2^ for both sexes after the age of 20–30 years according to the NKF guidelines [25]. In chronic kidney disease (CKD) patients, the average decline rate is 1–2.5 ml/min/1.73 m^2^ [26]. In patients with additional comorbidities in addition to CKD, the decline rate has been observed to be 2.16 and 2.07 ml/min/1.73 m^2^ per year in men and women, respectively [27]. In patients with both AF and CKD, the incidence of progression to end-stage renal disease is substantially higher than that in patients without AF [7]. Previous studies have shown that CKD patients free from AF after RFCA had better renal function [19, 21, 22]. Our study, on the other hand, showed that patients with mildly impaired renal function (eGFR of 60 to 90 ml/min/1.73 m^2^) could achieve improved renal function even with atrial arrhythmia recurrence after RFCA. Moreover, the absolute *△*eGFR has a closer association with the *△*CHA_2_DS_2_-VASc score and the type of recurrent atrial arrhythmia than the initial CHA_2_DS_2_-VASc score or AF type, which presumptively could be partially attributed to the reduced AF burden after RFCA.

Cardiovascular diseases and CKD are concordant. The results from previous studies indicate that the treatment of concomitant cardiovascular diseases could slow down the progression of renal function deterioration. The mainstay to slow down the progression of renal function deterioration is to control comorbidities, such as heart failure, hypertension, and diabetes mellitus [28]. As a practical tool to stratify stroke risk in NVAF patients, the CHA_2_DS_2_-VASc score is highly recommended for use by existing guidelines [29]. This score is the sum of cardiovascular risk factors that a patient may have. Stroke risk in NVAF patients has recently been considered a dynamic process due to increases in age and other new incident risk factors. Therefore, the change in score between baseline and follow-up, which was reflected by *△*CHA_2_DS_2_-VASc, could have greater value in predicting ischemic stroke [30]. Our study shows that a lower *△*CHA_2_DS_2_-VASc score was a significant factor associated with improved renal function, which suggests that controlling comorbidities should still be of great importance even after RFCA.

The AF burden, which means the amount or quantity of AF a patient has, is often expressed as the percent time of atrial fibrillation divided by total monitoring time, representing the duration patients are in an AF state. AF burden is an emerging parameter that has a close relationship to cardiovascular outcomes [31, 32]. In addition, some studies suggest that a higher AF burden could increase the risk of stroke [33].

Catheter ablation is considered to be an effective treatment for reducing AF burden [12, 34]. However, the endpoint of successful AF ablation has long been reflected as no atrial arrhythmia lasting longer than 30 s captured by using a monitoring device. Based on this threshold, the cumulative recurrence rate at 5 years after AF ablation ranges from 60% to 73% [35]. Recently, after the results of the CABANA study were released, the adoption of catheter ablation for AF patients has been questioned [10]. The crux of the argument focused on whether this binary evaluation of AF recurrence could reflect the total advantages of catheter ablation. In the CASTLE AF study, the AF recurrence rate in the ablation group was 36.9% after 60 months of follow-up. Furthermore, the AF burden was reduced from 51% to 20% in the ablation group but remained at more than 50% in the pharmacologic group at the 12-month follow-up [12]. In addition, the post hoc analysis of the CABANA trial showed that AF burden decreased by 69–88% in the ablation group and 48–73% in the medical therapy group after 5 years (*P* < 0.01) [17]. In the CIRCA-DOSE trial, the one-year success rate of AF ablation was 53%, and the AF burden was relatively reduced by nearly more than 98% [36].

It is unclear whether AF burden has a relationship with renal function impairment. However, several studies have shown that maintaining sinus rhythm is associated with better renal function, which implies that patients with renal dysfunction might benefit from reduced AF burden [19, 21, 22]. To evaluate the effect of AF burden on renal function, the pattern of arrhythmia change before and after the index ablation was classified into 4 groups in our study. The recurrence of paroxysmal or nonparoxysmal arrhythmia could roughly represent different AF burdens. Based on the results from our study, for AF patients with a reduced eGFR, considering the protection of renal function, clinicians should pay more attention to heart rhythm monitoring and timely conversion to sinus rhythm with medical therapy or catheter ablation. Moreover, our study provides another angle to look at the benefits of AF catheter ablation. In patients with mildly impaired renal function, even those with recurrence, after a mean follow-up of 11 months, renal function could still be improved after catheter ablation. This could be informative for clinicians referring patients for treatment. AF ablation could lead to a better outcome by reducing AF burden instead of eliminating AF completely in a selective group of patients.

With the increasing recognition of the importance of anticoagulation therapy in AF patients, there have been concerns of the effect the oral anticoagulants have on renal function. In a recent study of the eGFR in AF patients with different anticoagulants, patients treated with NOACs showed a lower decline compared to those treated with warfarin [37]. But, in our study, anticoagulation therapy failed to reach significance in the Cox regression, which might relate to the small sample size.

Our study had some limitations. The small sample size and heterogeneity of the ablation strategy due to difference in the left atrial substrate may prevent us from reaching a definite conclusion. Second, although we demonstrated the relationship between changes in eGFR and recurrent arrhythmia types, we were not able to verify how AF burden changes would affect renal function due to a lack of precise data on AF burden. Third, renal function in this study was evaluated by eGFR alone. Other measurements of renal function, such as proteinuria, were not used in this study. Fourth, this is a single-arm self-control study that aimed to reveal the change in renal function before and after RFCA. No real control group was recruited in this study. Fifth, the impact of early or late scheduled reablation on the change in eGFR could not be assessed, since all patients were scheduled to have a repeat session immediately after the recurrent AF in this study. Finally, the increase in eGFR was modest, and the clinical effect of the eGFR change needs further investigation.

## 5. Conclusions

In conclusion, in NVAF patients with mildly impaired renal function, even those with recurrence after the initial catheter ablation, we observed improvements in renal function, which was associated with a lower *△*CHA_2_DS_2_-VASc score and paroxysmal arrhythmia.

## Figures and Tables

**Table 1:** Clinical characteristics at baseline during the initial admission before RFCA.

*N* = 132	
Age (years)	62.8 ± 0.7
Men, *n* (%)	88 (66.7)
AF type- paroxysmal, *n* (%)	72 (54.5)
AF duration (months)	75.1 ± 6.5
Comorbidities	
Congestive heart failure, *n* (%)	2 (1.5)
Hypertension, *n* (%)	81 (61.4)
Diabetes mellitus, *n* (%)	11 (8.3)
Stroke, *n* (%)	7 (5.3)
Vascular disease, *n* (%)	0
Coronary artery disease, *n* (%)	1 (8.3)
Cardiovascular ICD, *n* (%)	4 (3.0)
CHA_2_DS_2_-VASc score	1.7 ± 0.1
Drug therapy	
ACEI/ARB, *n* (%)	62 (47.0)
*β*-blockers, *n* (%)	47 (35.6)
CCB, *n* (%)	29 (22.0)
Diuretics, *n* (%)	13 (9.8)
OAC-warfarin, *n* (%)	59 (44.7)
OAC-NOACs, *n* (%)	33 (25.0)
eGFR at baseline	78.0 ± 0.7
Echocardiographic parameters	
LVEF (%)	64.8 ± 0.4
LAD (mm)	39.0 ± 0.5
LVDd (mm)	47.2 ± 0.5

Continuous variables are expressed as the mean ± SD or median and interquartile range (IQR) if nonnormally distributed. Categorial variables are presented as numbers (percentages). AF, atrial fibrillation; ICD, implantable electronic device; ACEI, angiotensin-converting enzyme inhibitor; ARB, angiotensin receptor blocker; CCB, calcium channel blocker; OACs, oral anticoagulants; NOACs, nonvitamin K antagonist oral anticoagulants; eGFR, estimated glomerular filtration rate; LAD, left atrium diameter; LVEF, left ventricular ejection fraction; LAD, left atrial diameter; LVDd, left ventricular diastolic diameter.

**Table 2:** Univariate and multivariable Cox regression to identify factors associated with improved renal function in AF patients with mild renal dysfunction after RFCA.

*N* = 132	HR	95% CI	*P*	HR	95% CI	*P*
Age (years)	0.98	0.95–1.01	0.269	0.97	0.95–1.00	0.069
Men	0.80	0.51–1.28	0.355			
AF type- paroxysmal	1.56	1.00–2.44	0.051			
AF duration (months)	1.00	1.00–1.00	0.435			
Congestive heart failure	0.77	0.11–5.58	0.799			
Hypertension	0.94	0.61–1.46	0.785			
Diabetes mellitus	0.88	0.38–2.03	0.765			
Stroke	1.08	0.65–1.80	0.755			
Coronary artery disease	0.93	0.43–2.02	0.852			
Cardiovascular ICD	0.89	0.22–3.63	0.871			
Hypertrophic cardiomyopathy	0.05	0.00–320.51	0.500			
CHA_2_DS_2_-VASc score baseline	0.94	0.78–1.13	0.512			
*△*CHA_2_DS_2_-VASc score	0.44	0.25–0.78	0.005	0.42	0.24–0.74	0.003
OAC-warfarin	0.94	0.52–1.69	0.828			
OAC-NOACs	0.76	0.43–1.32	0.325			
ACEI/ARB	0.92	0.60–1.42	0.700			
*β*-blockers	0.95	0.60–1.49	0.809			
CCB	0.70	0.40–1.22	0.204			
Diuretics	0.61	0.27–1.41	0.250			
eGFR baseline	1.01	0.98–1.04	0.406			
LVEF	1.00	0.96–1.05	0.855			
LAD	0.95	0.91–0.99	0.017	1.00	0.95–1.05	0.912
LVDd	0.99	0.95–1.03	0.717			
Arrhythmia recurrence type- paroxysmal	2.64	1.50–4.62	0.001	2.97	1.60–5.48	0.001

AF, atrial fibrillation; ACEI, angiotensin-converting enzyme inhibitor; ARB, angiotensin receptor blocker; CCB, calcium channel blocker; OACs, oral anticoagulants; NOACs, nonvitamin K antagonist oral anticoagulants; LAD, left atrium diameter; LVEF, left ventricular ejection fraction; LAD, left atrial diameter; LVDd, left ventricular diastolic diameter; HR, hazard ratio; CI, confidence interval.

## Data Availability

Data are available on request from the authors.
